# Colorimetric Analysis of Ochratoxin A in Beverage Samples

**DOI:** 10.3390/s16111888

**Published:** 2016-11-10

**Authors:** Diana Bueno, Luis F. Valdez, Juan Manuel Gutiérrez Salgado, Jean Louis Marty, Roberto Muñoz

**Affiliations:** 1 Bioelectronics Section, Department of Electrical Engineering, CINVESTAV-IPN, Mexico City 07360, Mexico; dbueno@cinvestav.mx (D.B.); fvaldez@cinvestav.mx (L.F.V.); mgutierrez@cinvestav.mx (J.M.G.S.); 2 Université de Perpignan Via Domitia, BAE, Building S 52 Av. Paul Alduy, Perpignan Cedex 66860, France; jlmarty@univ-perp.fr

**Keywords:** model color, OTA, beer, wine, HSV, CIELab, RGB, YCbCr, CIEXYZ

## Abstract

This manuscript describes the use of a portable and low cost fluorescence setup to quantify the concentration of ochratoxin A (OTA) in beverage samples using an in-house developed system and different color models. It is reported that OTA is naturally fluorescent, for that reason an ultraviolet light at 365 nm was used to excite the samples and a Complementary Metal Oxide Semiconductor (CMOS) sensor was used to get a photograph of the OTA under excitation conditions, which is controlled by an executable interface designed in MATLAB. For each concentration of OTA, the coordinates with respect to each model color were obtained and plotted to quantify the mycotoxin present in the sample. It was possible to observe that despite the fact no extraction column was employed, the Red, Green, Blue (RGB) model shows a proportional relation to the evaluated concentrations. Despite the fact more analysis and other methods are required to quantify the OTA concentration, the brightness and a,b for the color-opponent dimensions (L*a*b) and Hue, Saturation, Value (HSV) tests provide results whereby it is possible to identify the concentration of OTA in beverage samples such as beer and wine.

## 1. Introduction

Food safety is important to prevent foodborne illnesses. There are many well-established analytical techniques like high performance liquid chromatography or gas chromatography for the analysis of food contaminants such as mycotoxins. Some limitations of these techniques are their high cost, lack of sensitivity and the need for skilled technicians, so there is a necessity for complementary techniques to detect quality parameters and safety threats in the rapid screening field. With the surge of innovative developments in photonics, new techniques for food analysis have emerged. 

Optical detection is one of the oldest and most established techniques [1], but it requires miniaturization for field measurements. Its advantages are simplicity of operation, low cost, low power consumption, and high stability. It is particularly appealing in food safety since it can detect analytes in complex matrices with minimal sample treatment and this detection method combines the selectivity of biology with the processing power of modern microelectronics and optoelectronics to offer powerful new analytical tools with major applications in the field of drug discovery, medicine, environmental and food processing industries. Biosensors in general and optical biosensors in particular show enormous potential for the detection of pathogens, pesticides, drug residues, heavy metals and other toxic substances present in foodstuffs [2]. 

Optical biosensors can benefit from advances in digital image processing or other types of signal processing techniques. Digital image processing is growing well beyond traditional “photographic” imaging. Chemical imaging takes advantage of a number of spectroscopic techniques. These techniques provide the needed information about the molecular composition, structure and dynamics [3,4,5].

Considering fluorescence and colorimetric detection in samples, the pixel is the measurement unit for the light emitted by a sample. Differences in the light may indicate the presence of a particular structure or substance. Knowing the value allows us to make comparisons and quantitative measurements. There are many color models to work with in image processing such as Red, Green, Blue (RGB), Hue, Saturation, Value (HSV), Hue, Intensity, Saturation (HIS), brightness and a,b for the color-opponent dimensions (CIE L*a*b) or tristimulus values (CIEXYZ). L*a*b units from RGB digital images were used to detect food appearance and color [6] and the RGB model combined with an image sensor was employed to detect mycotoxins [7,8].

Mycotoxins are secondary metabolites produced by fungi that are capable of causing disease and death in humans and other animals. One of the most important and most commonly occurring mycotoxins is ochratoxin A (OTA) [9]. OTA emits fluorescence under ultraviolet light [10] and it has been classified by the International Agency of Research in Cancer (IARC) as a class 2B carcinogen [11]. The main contributors to human OTA exposure are cereals, wine, coffee and beer [12,13,14]. The maximum permitted levels for wine is 2 µg/L and the level in beer has been established at 3 µg/L [13].

Considering the importance of detecting OTA in beverage samples and the use of the color models as an alternative detection method, a low cost and portable fluorescence detection device for the analysis of OTA was employed [8] to exploit color models in connection with image sensors to design colorimetric assays for the detection of the fluorescence of OTA avoiding the use of extraction columns. The data were processed in a graphical friendly-user interface designed in MATLAB^®^ R2011a. This is a first application of the use of different color models to detect OTA in wine and beer samples and the results were compared and validated using fluorescence detection. 

## 2. Materials and Methods 

Spectrophotometric cuvettes were purchased from Ratiolab (Dreieich, Germany). The sodium bicarbonate (NaHCO_3_) and methanol for analytical use were purchased from Sigma Aldrich (Saint-Quentin-Fallavier, France). Beer and rosé wine samples were obtained from a local supermarket (Perpignan, France), only one sample of beer and wine was employed. A sterile 0.45 µm syringe filter, and a reliable filtration system were purchased from Sartorius Stedim Biotech (Aubagne, France) and Ochratoxin A was purchased from Trilogy Lab (Washington, DC, USA).

### 2.1. Solutions and Measurements

A stock standard solution of 1 mg/mL was prepared by dissolving OTA (5 mg) in methanol (5 mL) and then storing it at −20 °C. It has been reported that OTA solutions in methanol stored at −20 °C are stable over a period of several years [15]. 

The calibration curve was obtained as follow: concentrations of 2, 5 and 10 ng/mL of OTA in methanol were prepared. Those were placed in the cuvette, excited with UV light and an image was taken and finally processed. The process to prepare the beverage samples was:

- *Blank*: 1 mL of methanol was mixed with 8 mL of the wine or beer sample and 1 mL of NaHCO_3_.
- *Concentrations*: 1 mL of the mix of 8 mL of sample along with 1 mL of 10% NaHCO_3_ and 1 mL of different previously prepared concentrations of OTA in methanol were mixed to get at final concentration of 2, 5 or 10 ng/mL.
- *Measurements*: In the device, the samples were excited with UV light, the blank was inserted and three images were taken, the sample was inserted two times more and the image was obtained. Finally, each sample was repeated. The samples of wine and beer were filtered using a 45 µm sterile syringe filter before spiking with OTA. All the measurements were made in triplicate.

### 2.2. Instruments 

A fluorescence instrument consisting of a Fluoroskan Ascent FL 2.6 instrument (Thermo Fisher Scientific, Espoo, Finland) equipped with Ascent software version 2.6. All the chemicals were weighed using a PB1501 scale (Metter Toledo, Columbus, OH, USA) with a precision of ±0.1 g. The ultraviolet (UV) emitter to 360–370 nm from NICHIA Corporation was supplied by Power Light Systems (Berlin, Germany). Electronic components were supplied by Farnell Element 14 (Chicago, IL, USA) and Mouser Electronics (Mansfield, TX, USA). Arduino^®^ UNO board, a serial port color camera module Link Sprit^®^ (LS-Y201-Infrared, Longmont, CO, USA) with a CMOS sensor and transistor-transistor logic (TTL) interface and the software employed was MATLAB R2011a^®^. 

#### Device Set-up Employed

**Figure 1:**
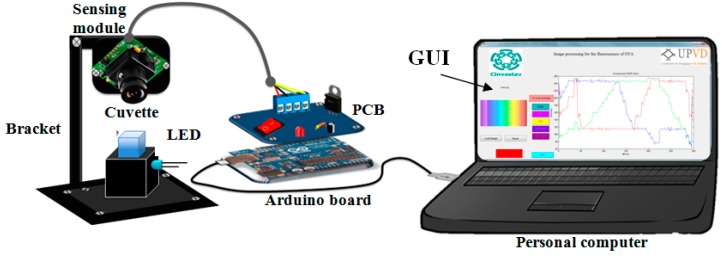
Sensing module connected to the final device and its control by the computer.

A low cost and portable fluorescence detection device for the analysis of OTA was employed (see Figure 1), by exploiting the color models in connection with the image sensors to design colorimetric assays for the detection of various analytes or compounds qualitatively and quantitatively without using an extraction column.

The portable fluorescence detection device is based on a bracket designed to hold the sensing module, which it is a serial port camera module and the cuvette in the chamber. One hole for the light emitter was drilled into s black poly-methyl-methacrylate cuvette holder at 90° from a serial port camera module, with a serial TTL interface, which captures Joint Photographic Experts Group (JPEG) images with a CMOS sensor from a serial port with communication via UART. The sensing camera required a 5 VDC power supply.

OTA shows fluorescence when it is excited with UV-light [16] and the fluorescence emitted has a linear relation with the concentration of the OTA. The UV-Light Emitting Diode (LED) used a voltage regulator (LM317) that is powered by a constant current through the USB port of an Arduino UNO board, employed as power source. 

The circuit was designed and placed in an electrolytic plate with dimensions of 30 × 20 mm^2^. In addition, the image was processed by automatic data processing graphical friendly-user interface (GUI) designed in MATLAB^®^ R2011a (see Figure S4 in the Supplementary Material) to obtain the color models that best matches with the concentration of the OTA in the wine and beer samples.

The image of every concentration was captured directly and processed following the technique or model color described in Section 3. An image is considered as a mathematical entity consisting of a spatial organization of pixels. Grey-scale or binary images are 2D arrays, which use a single-channel color space that is either limited to a 2-bit (binary) or intensity (grey-scale) color space. In contrast, true-color images are 3D arrays that corresponding to the red, green and blue components [17,18].

## 3. Results and Discussion

Before processing the images, each one was filtered using a smoothing filter, in order to reduce noise or effects that can occur as a result of the capture process, scanning and transmission. One example is the linear filter of the measurements. From the original image, the procedure generates a new image, whose intensity for each pixel is obtained by averaging the intensity values of the pixels in the original image, included in a predefined neighborhood environment. Finally, a convolution operation is performed between the image to be filtered and a mask [19,20].

### 3.1. Definition of the Colorimetric Methods Employed

The histogram of a gray scale image presents the relative frequency of occurrence of the various levels of the image. The histogram for an image in gray scale with intensities in the interval [0, *K*−1] produces a histogram H with *K* different values, a common image in gray scale of 8 bits is H = 2^8^ = 256. Every value is defined as h(i) = a, the number of pixels of I with the value of the intensity I for 0 ≤ i ≤ *K*. Finally, a vector one-dimensional h with a length of *K* is obtained and plotted [17,21]. Converting a color image to gray scale and displaying a gray scale histogram allows knowing the intensity of the histogram, so this technique was used to get the histogram in gray-scale.

The cumulative histogram H(i) consisted of a variation of the histogram, is defined as $H\left(i\right)=\sum_{j=0}^{i}h\left(j\right)$, for 0 ≤ i ≤ *K*, where the value of H(i) is the sum of the inferior values of the value specific given by (i) of the histogram h(j) with values j = 0,…,1. It is a monotonous and increasing function, with a maximum value $H\left(K-1\right)=\sum_{j=0}^{K-1}h\left(j\right)$. These calculations were programmed in Matlab to get the cumulative histogram (represented in the Figure S1 in the Supplementary Materials) for the beverage samples employed [22,23].

Improving the contrast of color images is a slightly more complex issue than for grey-scale intensity images. In this case, the application of histogram is the same as for the gray scale histogram, but the histogram is calculated for every channel (R, G, B), separately. Visual inspection of an image histogram can reveal the basic contrast that is present in the image and any potential differences in the color distribution of the image foreground and background scene components [17].

#### 3.1.1. RGB and HSV Models

Even though the RGB model is simple, it is one of methods most associated to the changes of color, RGB images are 3D arrays that are considered as three different 2D planes, one corresponding to each of the red (R), green (G) and blue (B) color channels. The colors present in a real image are nearly always a blend of color components from all three channels. If we consider that all the colors are represented with their RGB, then the RGB color space is essentially a 3D color space (cube) with axes R, G and B. The color black occupies the origin of the cube (position 0,0,0), corresponding to the absence of all three colors; white occupies the opposite corner (position 1,1,1), indicating the maximum amount of all three colors. The RGB color space based upon the portion of the electromagnetic spectrum visible to humans was described previously.

For the use of a simplified RGB color model that is optimized and standardized towards graphical displays, however, the principal problem with RGB is that it is perceptually nonlinear; it means that moving in a given direction in the RGB color cube does not necessarily produce a color that is perceptually consistent with the change in each of the channels. RGB space is inherently difficult for humans to comprehend because it is not related to the natural way we perceive colors. As an alternative, we may use perceptual color representations such as HSV.

Perceptual color space is an alternative way of representing true color images like the human perception and understanding of color besides of the RGB representation. The changes of the Hue, Saturation and Value (HSV) color space follow a perceptually acceptable color gradient. From an image analysis perspective, it allows the separation of color from lighting to a greater degree. A RGB image can be transformed into an HSV color space representation. Each of these three parameters can be interpreted as follows:

H (hue) is the dominant wavelength of the color (red, blue, green).
S (saturation) is the purity of color (in the sense of the amount of white light mixed with it).
V (value) is the brightness of the color (also known as luminance).

In MATLAB, HSV implementation each of H, S and V bounded within the range 0–1 by examining the individual color channels of images in the HSV space, the image objects are more consistently contained in the resulting hue field than in the channels of the RGB representation, despite the presence of varying lighting conditions over the scene. As a result, HSV space is commonly used for color-based image segmentation using a technique known as color slicing.

A portion of the hue color is isolated as the color range of interest, allowing objects within that color range to be identified within the image. This ease of color selection in HSV color space also results in its widespread use as the preferred method of color selection in computer graphical interfaces and as a method of adding false color to images. For the conversion of RGB to HSV, the follow equations are considered to do the conversion:

(1) Vmax=maximum(R,G,B)

(2) Vmin=minimum(R,G,B)

(3) V=Vmax−Vmin

(4) $$S=\{\begin{array}{c} \frac{V}{\text{Vmax}}\;\text{if Vmax}\;>0\\ 0\;V=0 \end{array}$$

(5) $$R^{\prime }=\frac{\text{Vmax}-R}{V}$$

(6) $$G^{\prime }=\frac{\text{Vmax}-G}{V}$$

(7) $$B^{\prime }=\frac{\text{Vmax}-B}{V}$$

(8) $$H^{\prime }=\{\begin{array}{c} B^{\prime }-G^{\prime }\;\text{if}\;R=\text{Vmax}\\ R^{\prime }-B^{\prime }+2\;\text{if}\;G=\text{Vmax}\\ G^{\prime }-R^{\prime }+4\;\text{if}\;B=\text{Vmax} \end{array}$$

(9) $$H=\frac{1}{6}*\{\begin{array}{c} \left(H^{\prime }+6\right)\;\text{if}\;H^{\prime }<0\\ H^{\prime }\;\text{otherwise} \end{array}$$

#### 3.1.2. YC_b_C_r_ Model

YC_b_C_r_ is a color model used in standardized images for television; in the case of the YC_b_C_r_ model, the chromatics components C_b_ and C_r_ are obtained as the difference between the luminance and the color plane B and R. The equations employed for the calculation of this color model are created from the corresponding gamma-adjusted RGB source using two defined constants $K_{b}$ and $K_{r}$ as follows:

(10) $$Y^{\prime }=K_{r}*R^{\prime }+\left(1-K_{r}-K_{b}\right)*G^{\prime }+K_{b*}B^{\prime }$$

(11) $$C_{b}=\frac{1}{2}\frac{\left(B^{\prime }-Y^{\prime }\right)}{1-K_{b}}$$

(12) $$C_{r}=\frac{1}{2}\frac{\left(R^{\prime }-Y^{\prime }\right)}{1-K_{r}}$$

where $K_{b}$ and $K_{r}$ are derived from the definition of the corresponding RGB space, the equivalent matrix manipulation is often referred to as the “color matrix”. Here, the prime′ symbol mean gamma correction is being used; thus R′, G′ and B’ and 0 representing the minimum intensity.

All color models mentioned previously are associated with physical measures of the device used to display the information. To generate color regardless of the device used, it is necessary to have a color model that does not consider the representation; these models are called colorimetric or calibrated.

#### 3.1.3. CIEXYZ Model

In 1920, the Comission Internationale de L’Eclairage (CIE) [24] defined a system of describing the color of an object based on three primary stimuli: red (700 nm), green (540 nm) and blue (430 nm), whereby all the colors appear as different combinations of these. The amounts of red, green and blue needed to form any given color are called the tristimulus values, X,Y and Z, respectively. A color is represented by a set of chromaticity coordinates or trichromatic coefficients, x, y and z as defined below:

(13) $$x=\frac{X}{X+Y+Z}\;;y=\frac{Y}{X+Y+Z}\;;\;z=\frac{Z}{X+Y+Z}$$

**Figure 2:**
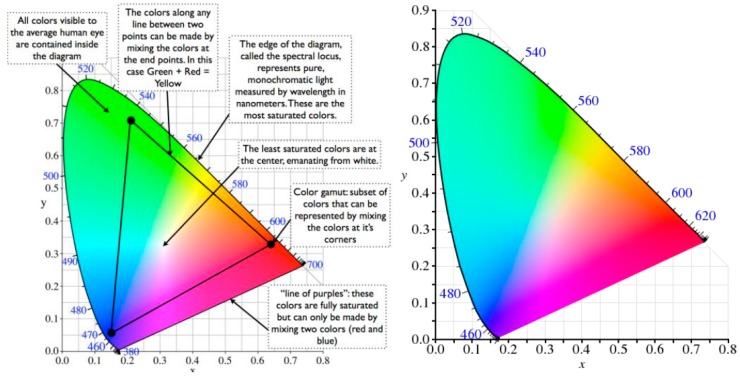
CIE chromaticity diagram.

The value x, y give the form of the CIE diagram, represented in Figure 2. It can be represented by Yxy where Y represents the luminance component of the XYZ system. A constant value of Y provides the horizontal plane of the CIE diagram. The points (x, y) along the border of the CIE surface are the spectral colors having the maximum value of saturation, besides different wavelength. With this, the position of each color can be calculated in relation to any primary color. The exception is connecting line which lies between 380 nm and 780 nm, it is out of the primary colors. In the halfway point through the CIE diagram increases the color saturation up to be a white point of the model, which is reached when x = y = 1/3 or X = Y = Z= 1.

#### 3.1.4. L*a*b* Model

The L*a*b model is defined by three variables, L* represents brightness and a* and b are for the hue component. The value of a* defines the distance along the red-green axis, while the b* represents the distance along the blue-yellow axis in the diagram CIE XYZ. The three components that define the space are relative to a white point Cref = (Xref, Yref, Zref), where a nonlinear correlation is used. The following is a brief description of how the CIE Lab values are computed from the reflectance value of an object. To take the tristimulus values X,Y,Z, we consider:

(14) $$L\;*=116{\left(\frac{Y}{Y_{n}}\right)}^{1/3}-16$$

(15) $$a\;*=500\left[{\left(\frac{X}{X_{n}}\right)}^{\frac{1}{3}}-{\left(\frac{Y}{Y_{n}}\right)}^{\frac{1}{3}}\right]$$

(16) $$b\;*=200\left[{\left(\frac{Y}{Y_{n}}\right)}^{\frac{1}{3}}-{\left(\frac{Z}{Z_{n}}\right)}^{\frac{1}{3}}\right]$$

X, Y and Z are the tristimulus values of the object measured.
X_n_, Y_n_ and Z_n_ are the tristimulus values of a reference white object.
L* is the visual lightness coordinate.
a* is the chromatic coordinate ranged approximately from red to green.
b* is the chromatic coordinate ranged approximately from yellow to blue.

### 3.2. Colorimetric Methods Applied to Beverage Samples Spiked with OTA

**Figure 3:**
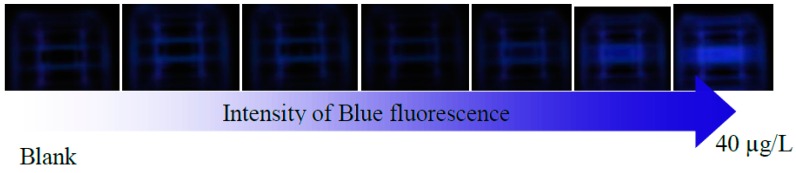
Increase of the fluorescence depends of the concentration of OTA in the sample.

**Figure 4:**
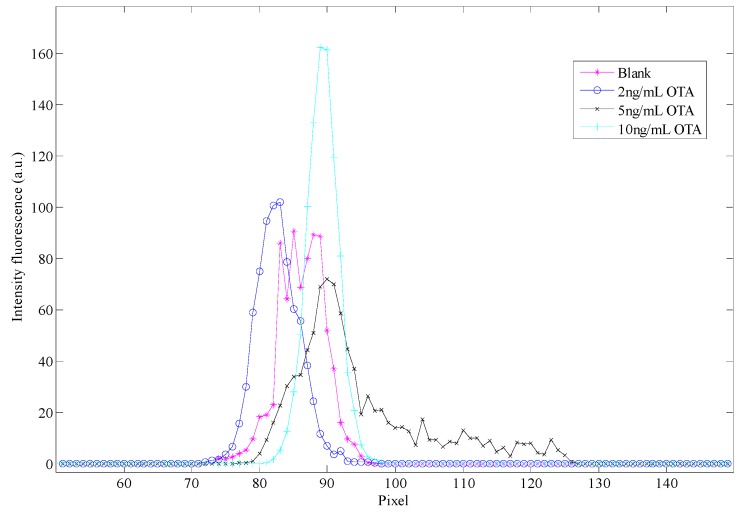
Gray scale histogram for the blank (sample of methanol) and specific concentrations of OTA prepared in methanol. This kind of graphic is called a calibration curve.

**Figure 5:**
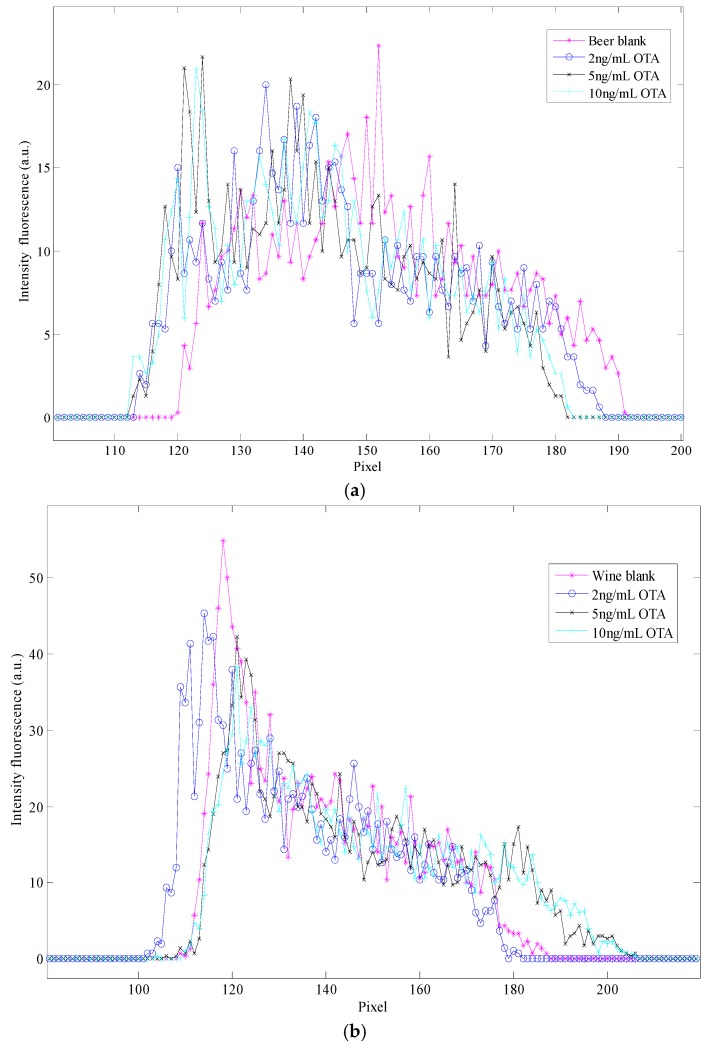
Gray scale histogram for (**a**) beer and (**b**) wine samples spiked at 2, 5 and 10 ng/mL OTA.

The variation of the light depending on the concentration of OTA is presented in Figure 3. The color images from spiked samples of beer and wine with OTA and concentration of OTA prepared in methanol were converted into a gray image in order to obtain their gray scale histogram; the calibration curve of that histograms is presented in the Figure 4, the beer and wine samples spiked with OTA are presented in the Figure 5. In Figure 4 the spectrum for the blank is wider than the range of the OTA concentrations and the higher OTA concentration is a narrow spectrum. For the biggest concentration, there is a maximum in the intensity of the light. 

With the gray scale histogram presented in the Figure 4 and Figure 5, was no possibility to differentiate between the concentrations of the beverage samples, however was possible to identify that they corresponded to two different samples, as in case of the beer samples the histogram shows a wide spectrum and the spectrum presents a decreasing slope in the wine samples.

With the cumulative histogram it was possible to see a difference between the concentrations. The concentrations were grouped in two groups, these are: (1) low concentrations (blank and 2 ng/mL) and (2) high concentrations (5 and 10 ng/mL). In addition, there is a slope but it does not show significant changes between concentrations. The results of the cumulative histogram and the decomposition of the image into its blue components for the calibration curve and the beverage samples spiked with OTA can be consulted in the Supplementary Materials (Figures S1 and S2).

In the case of the beer samples spiked with OTA, is possible to differentiate the blank from samples spiked with OTA, and shifting leftward that is to a greater concentration of OTA, the slope appears slightly shifted to the left, although the slope values for each concentration show no significant changes. For the wine samples, the results are grouped in low or high concentrations as in the case of the calibration curve.

The histogram of the blue component for the calibration curve of each concentration is moved to the right; this indicates that the main light colors are presented as the intensity of the OTA fluorescence. The histogram of the blue component presents the same behavior that the gray scale histogram and the histogram is presented in Figure S2 of the Supplementary Materials.

Table 1, Table 2 and Table 3 present the mean of the color model employed together the beverage samples spiked with OTA. Table 1 presents the mean of each color model employed in the colorimetric analysis of the calibration curve for the detection of OTA.

**Figure 6:**
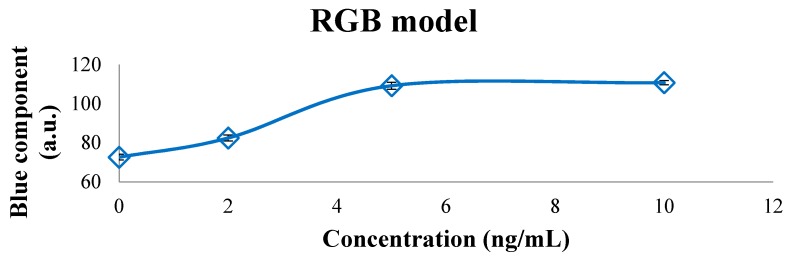
Blue component of the RGB model for the calibration samples. The concentrations of OTA show a proportional relation with its blue component.

Figure 6 shows that the blue component of the RGB model has a proportional relation with the concentrations, except for 10 ng/mL where, it shows that the image sensor is saturated, so it is very approximate value of the previous concentration, as well as V component of the HSV model and b* component from L*a*b* model present a proportional relation from the concentrations and the saturation of the image sensor for the last concentration. 

A representation of the mean of color models employed in the colorimetric analysis of beverage samples spiked with OTA such as beer is given in Table 2 and for the wine in Table 3.

**Figure 7:**
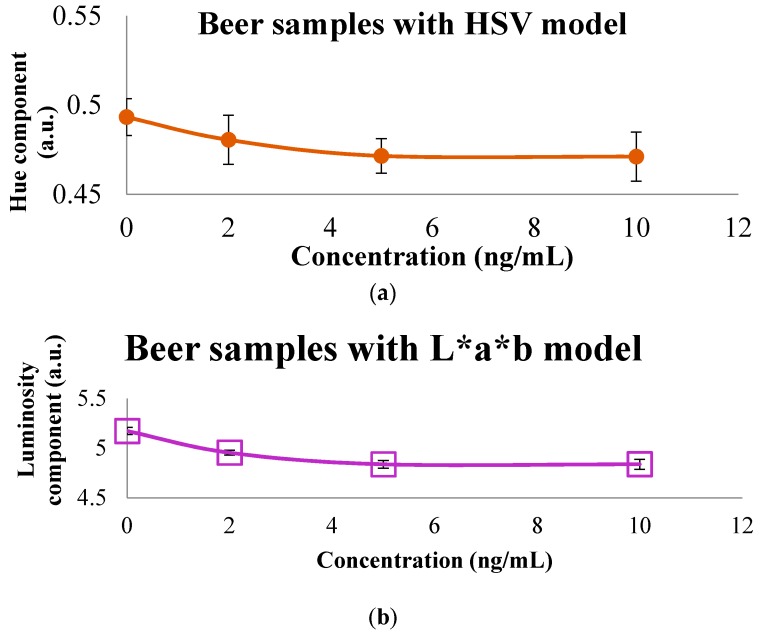

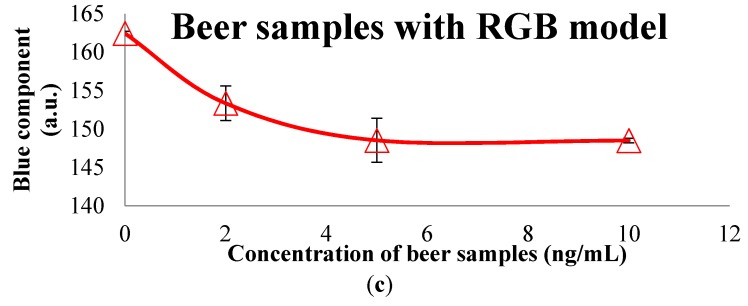
Color models such as (**a**) HSV, (**b**) L*a*b* and (**c**) RGB of beer samples spiked with OTA that presents a proportional relation between the components.

**Figure 8:**
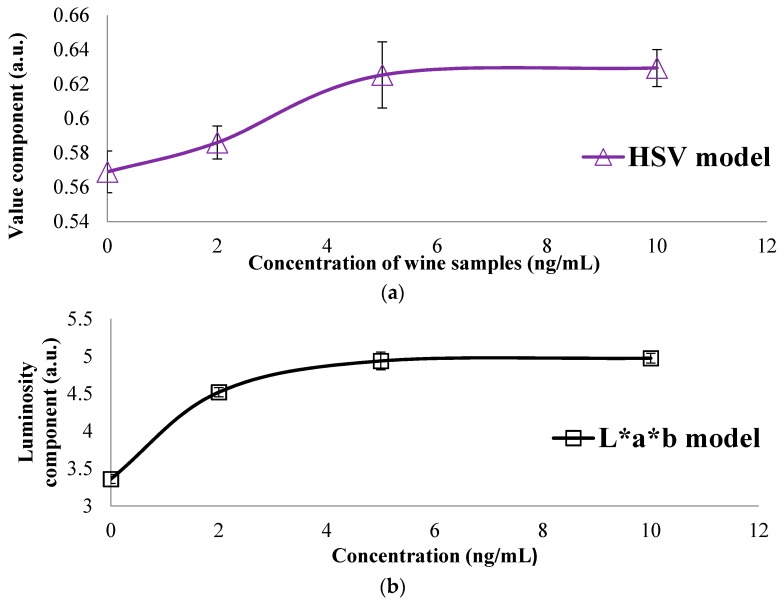

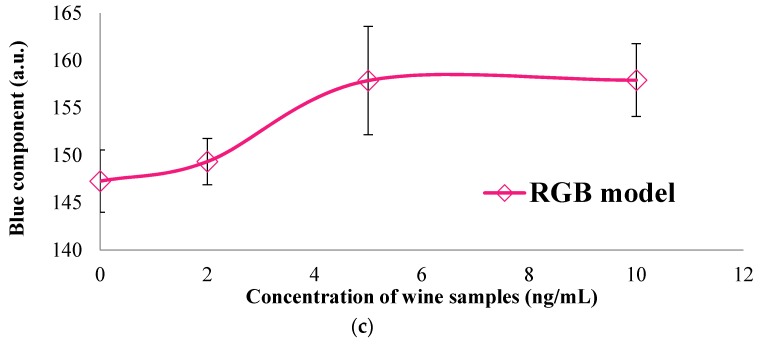
Color models employed for wine samples spiked with OTA are (**a**) HSV; (**b**) L*a*b* and (**c**) RGB that presents a proportional relation between the components.

The graphic representation of the color models presented in Table 2 is shown in Figure 7, where a similar result as for the last concentration was obtained, compared with the calibration curve. In spite of the fact that different components provide a proportional relationship for each concentration for the HSV, L*a*b and RGB models, this is presented in the Table 3, where the value of the different models employed with wine samples is summarized, and HSV, L*a*b and RGB models present a proportional relation with the evaluated concentration as seen in Figure 8. For example, for a major concentration, less value of the component is an indirect relation. A direct relation exists between major concentrations with the major value of the component evaluated.

Table 3 summarizes the mean of the different models employed to detect the concentration of OTA in wine samples, where the color models that show a relation such as HSV, L*a*b and RGB of samples spiked with OTA are presented in Figure 7.

**Figure 9:**
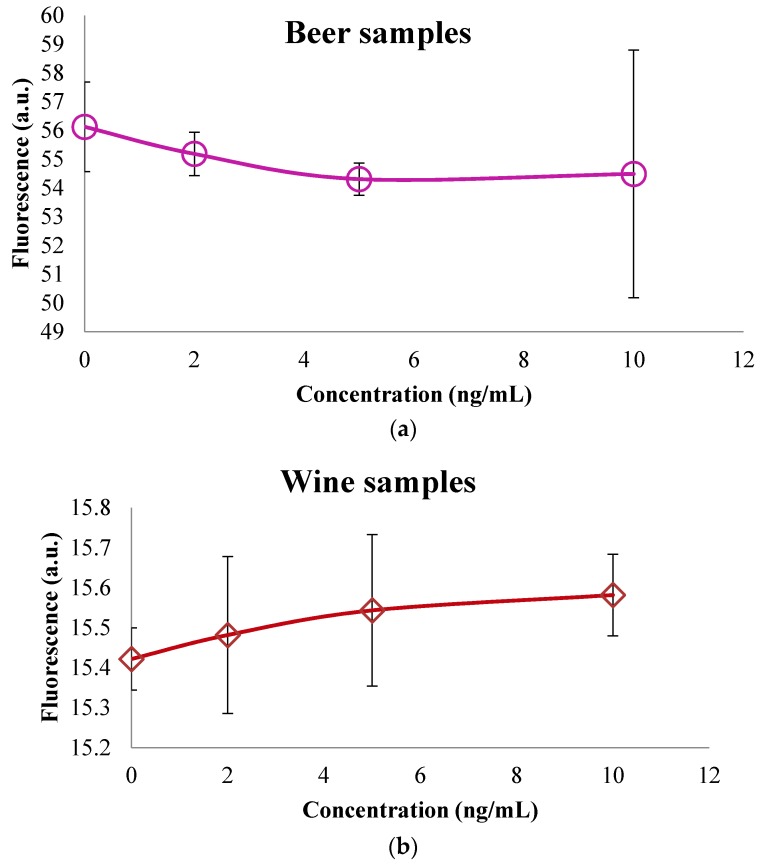
Fluorescence of the (**a**) beer samples spiked with OTA and for the (**b**) wine samples.

The results were compared with a conventional fluorimeter; the results are presented in Figure 9. A proportional relation for the wine samples and the samples of beer with the fluorimeter result was observed. This behavior is similar to the results obtained with the developed device. The values of the fluorescence for each concentration of wine and beer, do not present significant differences.

Table 4 shows the differences between the developed device and conventional equipment or other developed devices. One more color model employed was to get the CIE XYZ diagram for each concentration, the images of the diagrams presented on the Supplementary Materials (Figure S3). There is no difference among the concentrations; however, the values obtained demonstrated that the emission spectrum was located at 440–460 nm depending on the sample. This wavelength corresponds to the excitation wavelength of OTA for samples prepared in methanol [15]. Steinbrück et al. suggested using a wavelength excitation/detection scheme at 380 nm/ 440 nm [16]. 

Table 4 shows a LOD of 2 µg/L for the developed system, a limit comparable with the limit of conventional equipment like HPLC. Finally the LOD (limit of quantification) is 5 µg/L. 

In addition the small dimensions and weight provide portability and the short time to take the image and process the results in an automatic way with a laptop and work with wine, beer or other samples, makes it possible to use the proposed device for in situ detection. The developed device is easy to transport, because of the sensing module with the camera and the cuvette are fixed to the bracket. Finally, the price of the developed device considering the electronic components of the electrolytic plate, the Arduino board, the serial camera and the black poly-methyl-methacrylate employed in the bracket, is about 60 USD.

## 4. Conclusions

There is a proportional relation of color models with the fluorescence intensity of different concentrations of OTA. Wine samples are more complex that beer samples because the color of the wine, so sometimes this is not a relationship between the spiked samples and the concentrations. With the proposed method and the developed instrumentation, it is possible to identify spiked samples with different concentrations of OTA employing a low cost and portable device without the need to use extraction columns. Different color models were employed to identify samples spiked with OTA, considering the results it was possible to choose the best color model for the detection of OTA. For future work we propose to use more than one method at the same time to identify the OTA concentrations. According to the obtained results more analysis and other methods are required to quantify the OTA concentration without the use of extraction columns. 

## Figures and Tables

**Table 1:** Calibration curve of the color model.

		Blank	2 ng/mL	5 ng/mL	10 ng/mL
**HSV**	**H**	0.6742	0.0000	0.6054	0.6190
	**S**	0.1744	0.0411	0.2056	0.2805
	**V**	0.3452	0.3837	0.4281	0.4342
**Lab**	**L**	2.9088	2.8184	3.2277	3.0343
	**a**	−0.6617	−0.5388	−0.6525	−0.5070
	**b**	0.3776	0.1251	−0.1919	−0.3332
**RGB**	**R**	87.6008	80.2075	85.6717	79.5945
	**G**	86.4510	82.2542	95.9654	88.3748
	**B**	72.5807	82.3941	109.1670	110.7179
**YCbCr**	**Y**	89.1806	86.1331	97.0433	91.8359
	**Cb**	121.7540	128.3479	135.2807	138.9271
	**Cr**	129.6457	126.9936	122.4297	122.6169

**Table 2:** Color model employed with the beer samples spiked with OTA.

		Blank	2 ng/mL	5 ng/mL	10 ng/mL
**HSV**	**H**	0.4933	0.4806	0.4716	0.4712
	**S**	0.3059	0.2807	0.2687	0.2774
	**V**	0.6565	0.6254	0.6090	0.6140
**Lab**	**L**	5.1720	4.9538	4.8370	4.8375
	**a**	−1.6932	−1.5951	−1.5425	−1.5571
	**b**	−0.1835	−0.1205	−0.0877	−0.1052
**RGB**	**R**	115.7367	114.0567	112.9867	79.5767
	**G**	167.2433	159.4667	155.3033	156.5533
	**B**	162.4400	153.3400	148.5233	148.5036
**YCbCr**	**Y**	145.9599	140.6992	137.8622	138.5479
	**Cb**	133.4952	131.9739	131.2191	131.7138
	**Cr**	105.7098	108.4872	109.9113	109.0998

**Table 3:** Color model employed with the wine samples.

		Blank	2 ng/mL	5 ng/mL	10 ng/mL
**HSV**	**H**	0.5208	0.5226	0.5027	0.5015
	**S**	0.2805	0.2736	0.2859	0.2859
	**V**	0.5689	0.5860	0.6252	0.6293
**Lab**	**L**	3.3598	4.5206	4.9374	4.9740
	**a**	−1.3657	−1.3097	−1.5539	−1.5833
	**b**	−0.2887	−0.2834	−0.2229	−0.1923
**RGB**	**R**	110.1233	107.0967	112.6433	113.4567
	**G**	148.5033	142.9267	158.4767	159.9333
	**B**	147.2698	149.3367	157.8900	157.8915
**YCbCr**	**Y**	134.2763	130.1840	140.2895	141.1679
	**Cb**	136.2398	136.0407	134.4983	133.6966
	**Cr**	110.7673	111.8285	107.8862	107.7507

**Table 4:** Comparison between the fluorescence detection of the developed device and other methods.

Analytical Features	HPLC	Developed Device	Smartphone as Detector [7]	Amorphous Silicon Photodiode [25]	Array Biosensors (Cereals & Beverage) [26]	Fluorometer for Aflatoxin Detection [27]
LOD (Limit of Detection)	2 µg/L	2 µg/L	2 µg/L	850 ng/L	Cereals (3.8 to 100 ng/L), coffee 7 ng/L and wine 38 ng/L	<0.025 ng/L
Analysis time	8 min	1 min	Less than one min	1–2 min	-	-
Dimensions without computer	≈340 × 400 × 1000 mm^3^	≈145 × 145 × 150 mm^3^	≈115.2 × 58.6 × 9.3 mm^3^	≈6.2 × 12.6 mm^2^	-	-
Weight	>34 Kg	<1 Kg	1.4Kg	<1 Kg	-	-
Sample reusability	Yes	No^a^	No^a^	No^a^	No^a^	No ^a^
Purpose	Q	Q or S	Q or S	Q	Q or S	Q or S
Portability	No	Yes	Yes	No^b^	Yes	Yes
Offline data processing	No	Yes	Yes	No	Yes	Yes

^a^ It is possible to use and confirm in analytical methods or other tests; ^b^ Require spectrophotometer or other conventional equipment; Q = Quantification; S = Scanning.

## Supplementary Materials

The following are available online at http://www.mdpi.com/1424-8220/16/11/1888/s1, Figure S1: Cumulative histogram for gray scale of (a) calibration curve (b) beer and (c) wine samples spiked at 2, 5 and 10 ng/mL OTA, Figure S2: Histogram of blue component (a) calibration curve (b) beer and (c) wine samples spiked with OTA, Figure S3: CIEXYZ diagrams of the tested samples: calibration, beer and wine spiked with OTA.
