# Supplementary material for: Colorimetric Analysis of Ochratoxin A in Beverage Samples
Source: Sensors (Basel). 2016 Nov 10;16(11):1888. doi: 10.3390/s16111888 (PMC5134547; doi:10.3390/s16111888)
Supplement: Supplementary file 1 [file sensors-16-01888-s001.pdf]

Supplementary Materials: Colorimetric Analysis of Ochratoxin A in Beverage Samples

Diana Bueno, Luis F. Valdez, Juan Manuel Gutiérrez, Jean Louis Marty and Roberto Muñoz

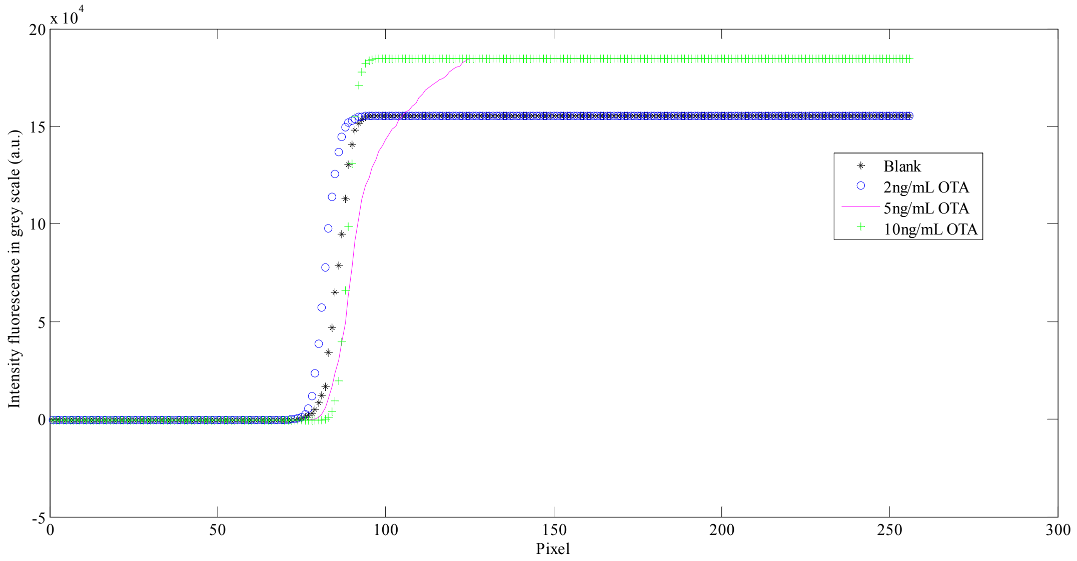

(**a**)

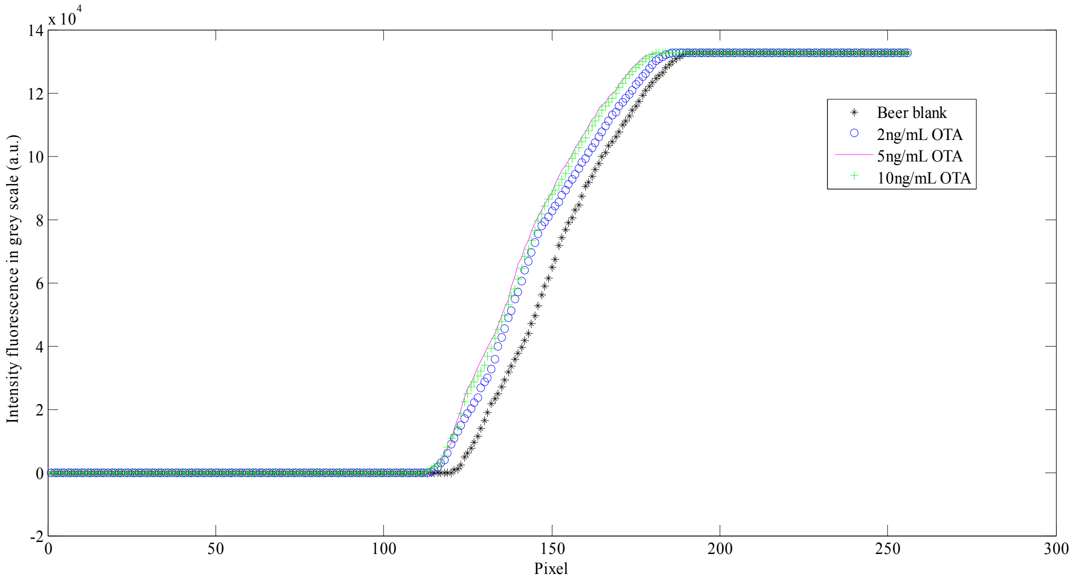

(**b**)

Figure S1. *Cont.*

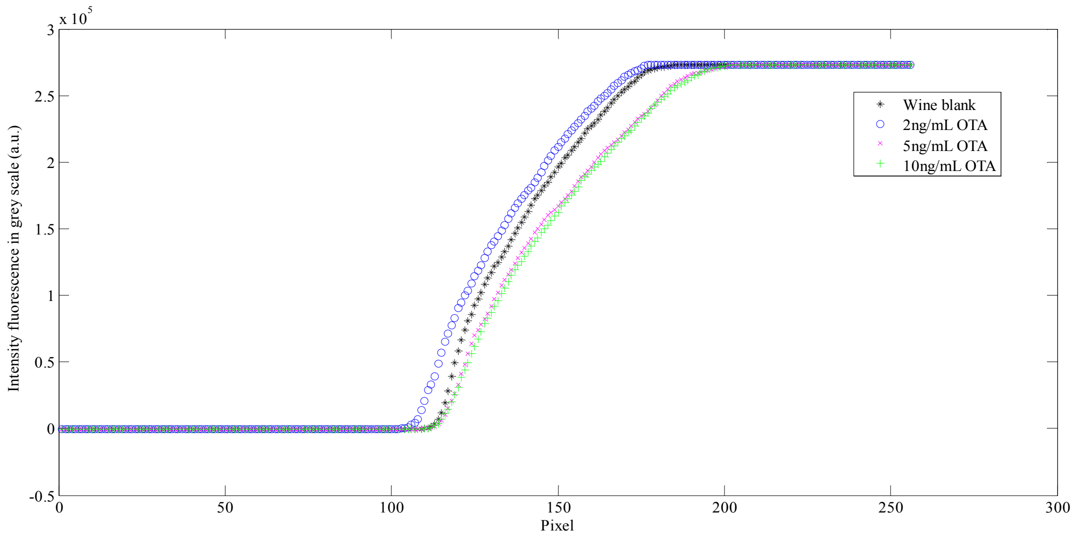

(**c**)

Figure S1. Cumulative histogram for gray scale of (**a**) calibration curve (**b**) beer and (**c**) wine samples spiked at 2, 5 and 10 ng/mL OTA.

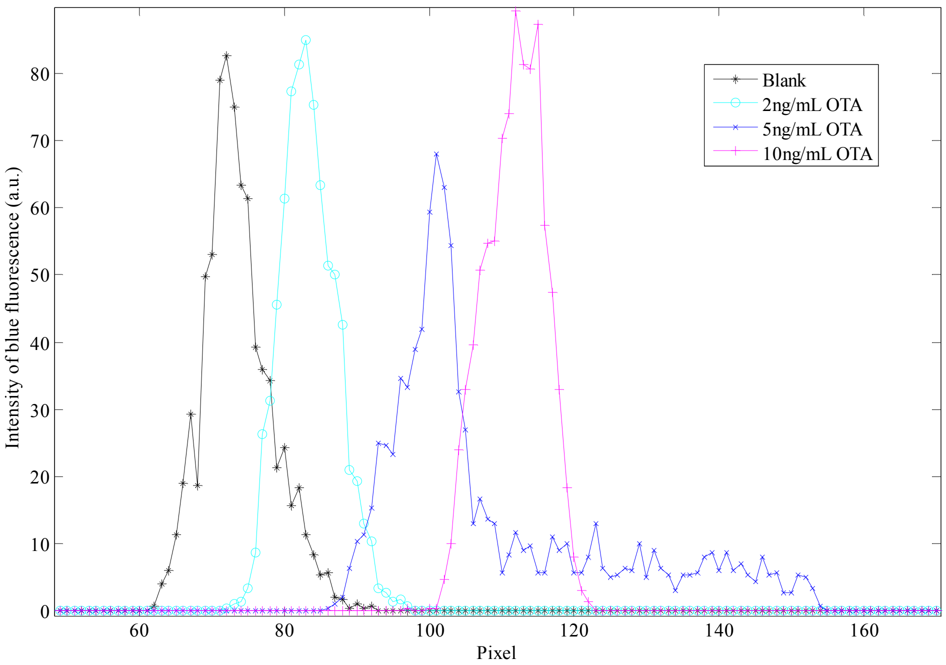

(**a**)

Figure S2. *Cont.*

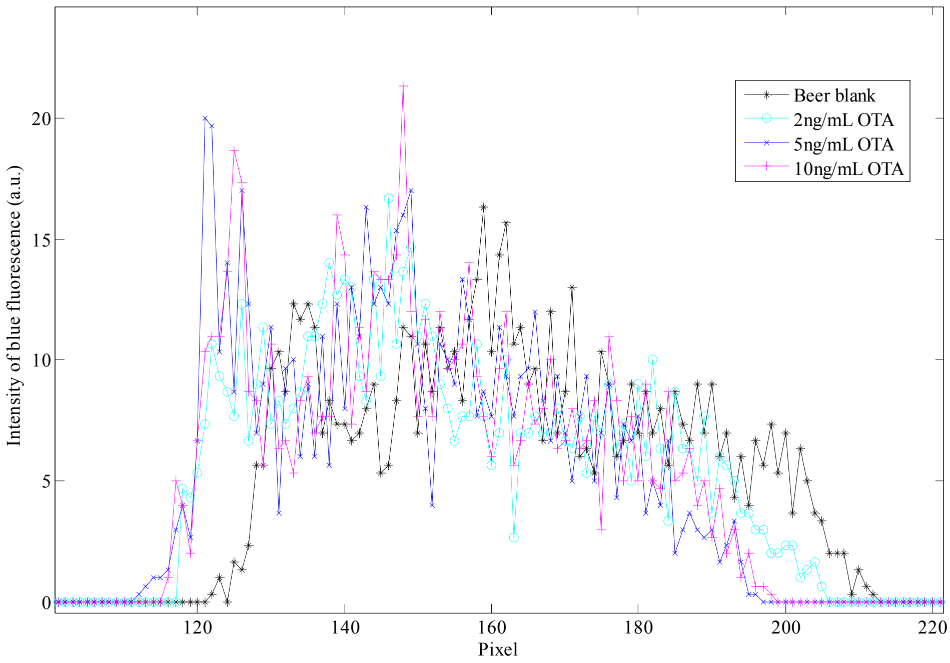

(**b**)

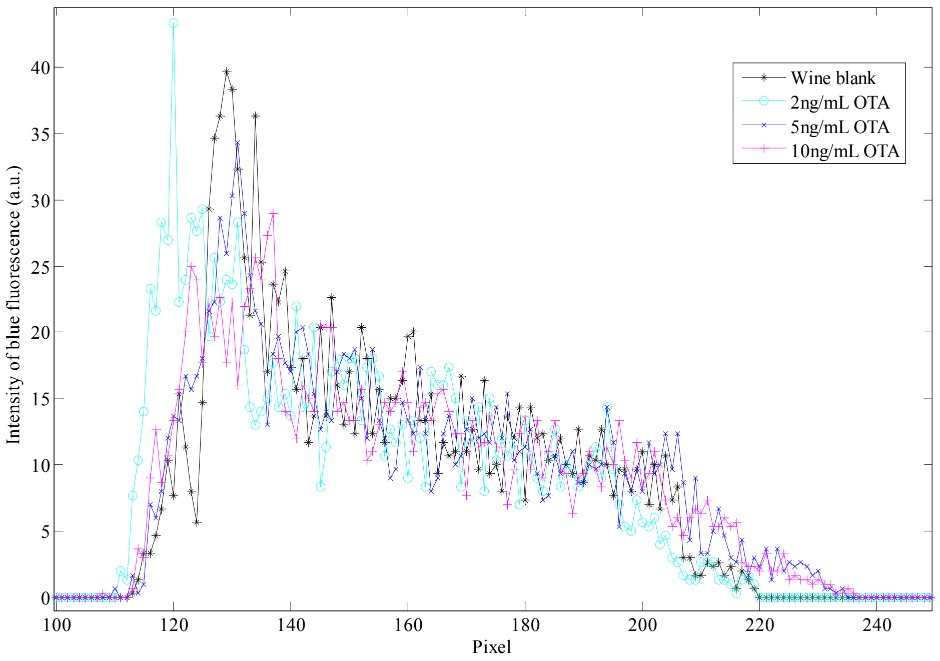

(**c**)

Figure S2. Histogram of blue component (**a**) calibration curve (**b**) beer and (**c**) wine samples spiked with OTA.

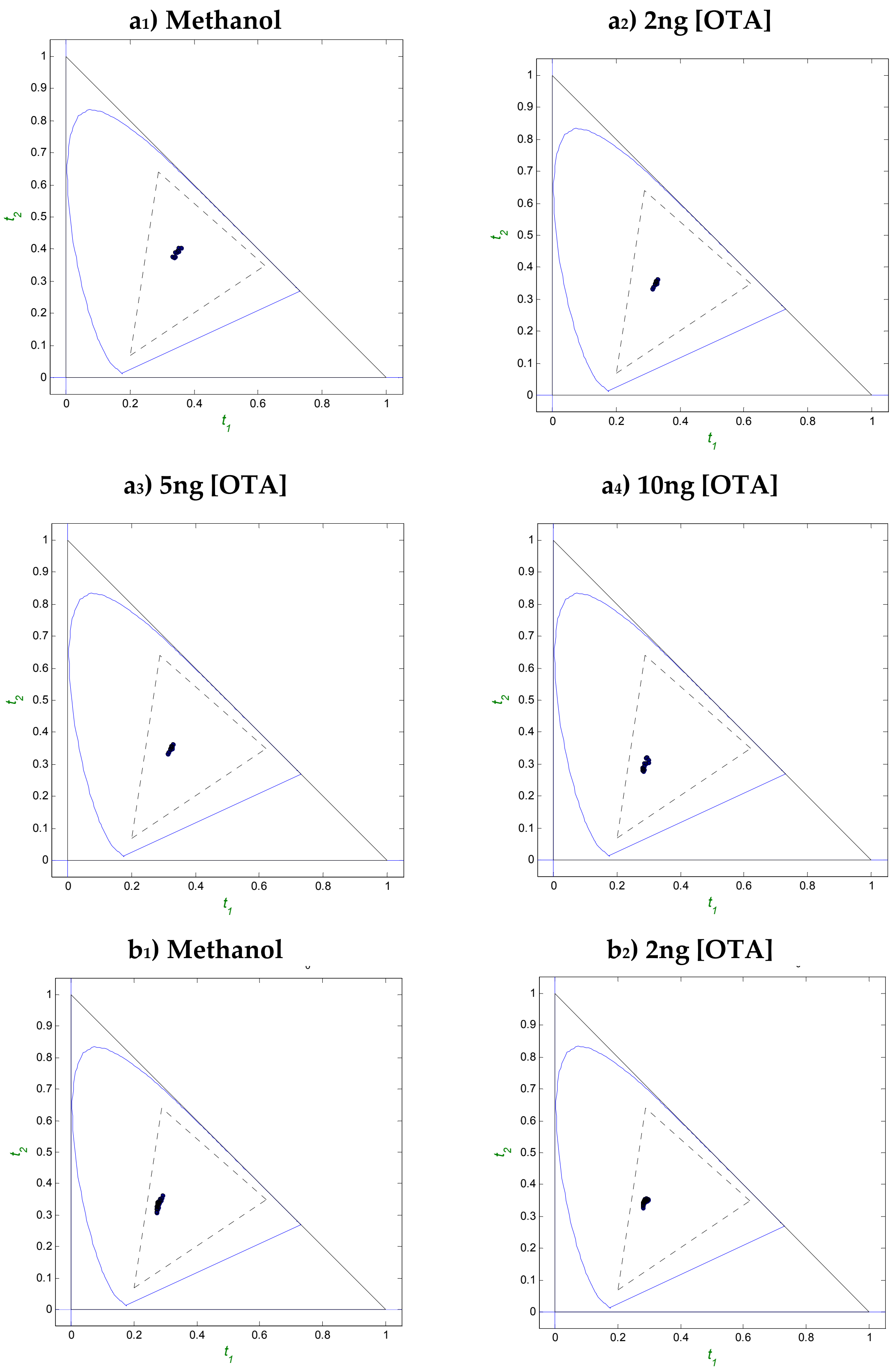

Figure S3. *Cont*.

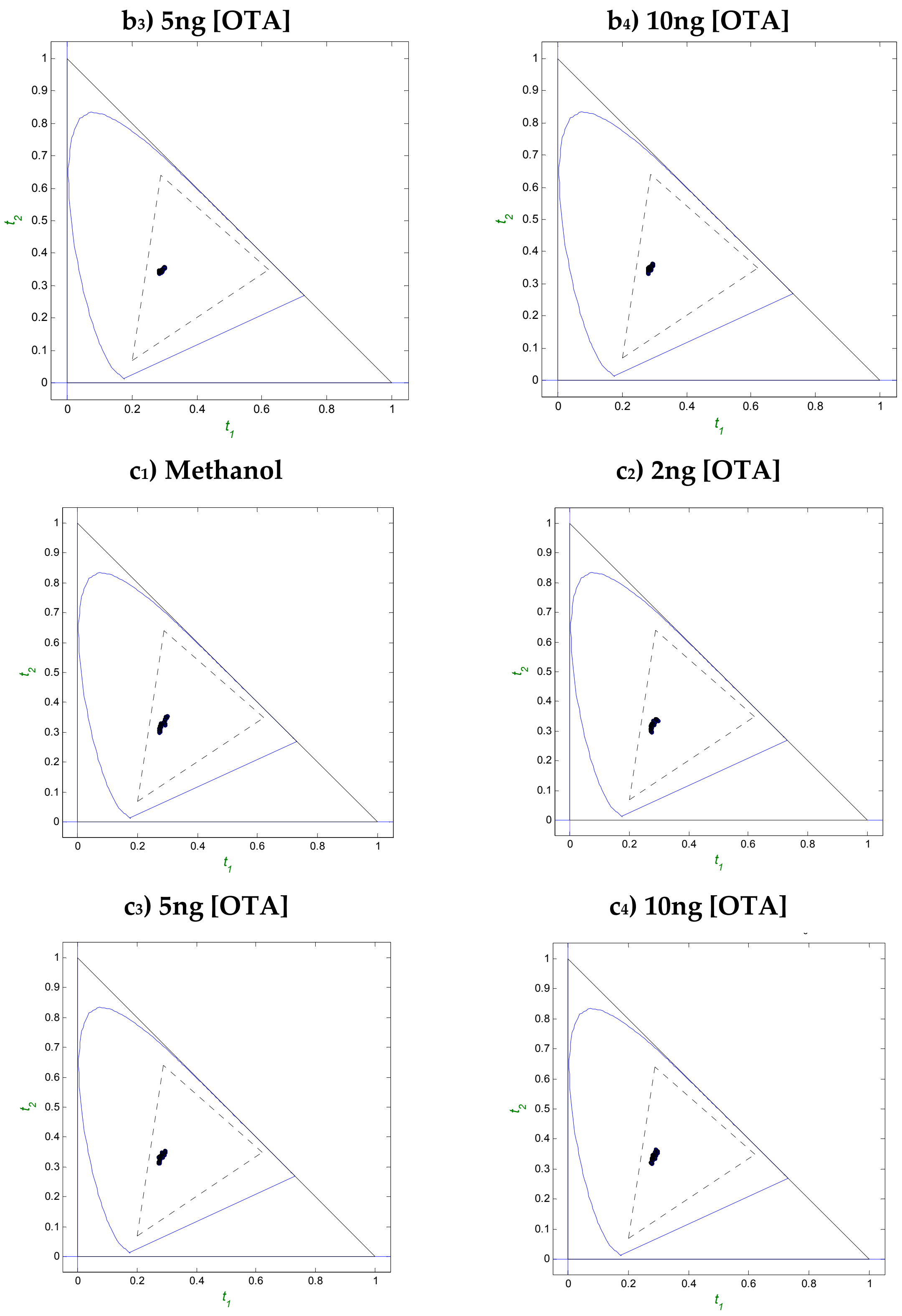

Figure S3. Diagram CIEXYZ of the samples tested. The calibration curve is represented in figures (**a**1–**a**4); figures (**b**1–**b**4) represents the beer spiked samples and figures (**c**1–**c**4) correspond to wine spiked samples at different OTA concentrations.

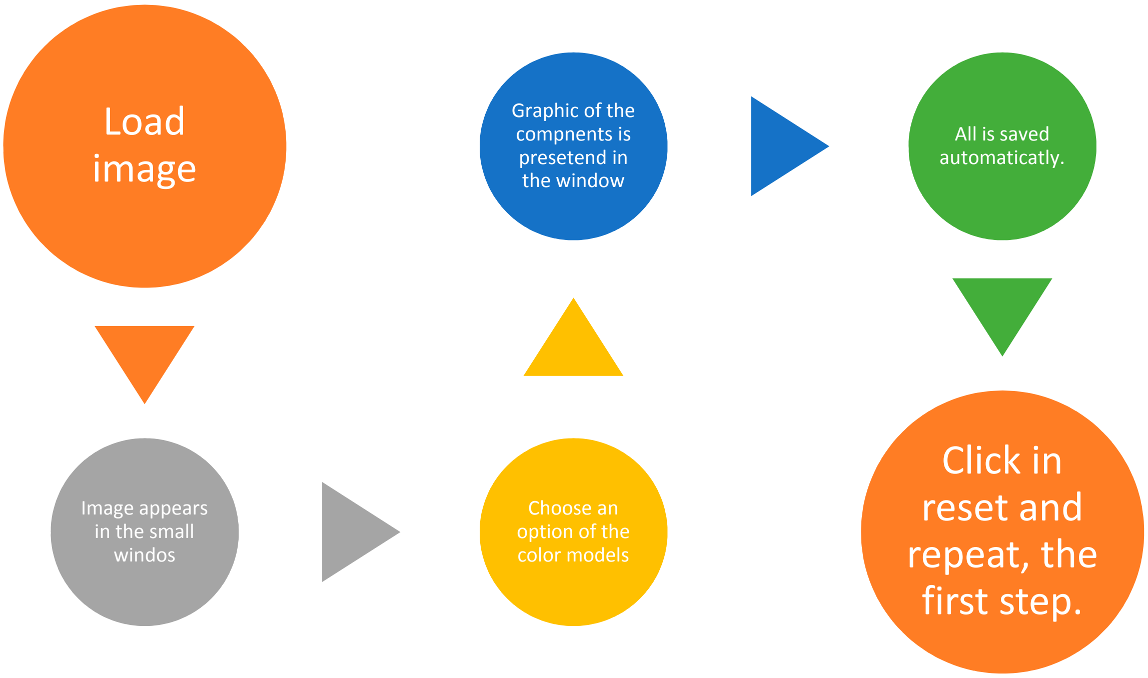

Figure S4. Block diagram of the graphical user interface (GUI) employed.
